# Targeted mutagenesis of the *CYP79D1* gene *via* CRISPR/Cas9-mediated genome editing results in lower levels of cyanide in cassava

**DOI:** 10.3389/fpls.2022.1009860

**Published:** 2022-10-26

**Authors:** Bicko Steve Juma, Asunta Mukami, Cecilia Mweu, Mathew Piero Ngugi, Wilton Mbinda

**Affiliations:** ^1^ Institute for Biotechnology Research, Jomo Kenyatta University of Agriculture and Technology, Nairobi, Kenya; ^2^ Pwani University Bioscience Research Centre, Pwani University, Kilifi, Kenya; ^3^ Department of Biochemistry, Microbiology and Biotechnology, Kenyatta University, Nairobi, Kenya; ^4^ Department of Life Sciences, South Eastern Kenya University, Kitui, Kenya; ^5^ Department of Department of Biochemistry and Biotechnology, Pwani University, Kilifi, Kenya

**Keywords:** cassava, CRISPR/Cas9, cyanide, *MeCYP79D1*, targeted mutagenesis

## Abstract

Cassava is the world’s most essential food root crop, generating calories to millions of Sub-Saharan African subsistence farmers. Cassava leaves and roots contain toxic quantities of the cyanogenic glycoside linamarin. Consumption of residual cyanogens results in cyanide poisoning due to conversion of the cyanogens to cyanide in the body. There is a need for acyanogenic cassava cultivars in order for it to become a consistently safe and acceptable food, and commercial crop. In recent years, the CRISPR/Cas system, has proven to be the most effective and successful genome editing tool for gene function studies and crop improvement. In this study, we performed targeted mutagenesis of the *MeCYP79D1* gene in exon 3, using CRISPR/Cas9, *via Agrobacterium*-mediated transformation. The vector design resulted in knockout in cotyledon-stage somatic embryos regenerated under hygromycin selection. Eight plants were recovered and genotyped. DNA sequencing analysis revealed that the tested putative transgenic plants carried mutations within the *MeCYP79D1* locus, with deletions and substitutions being reported upstream and downstream of the PAM sequence, respectively. The levels of linamarin and evolved cyanide present in the leaves of *mecyp79d1* lines were reduced up to seven-fold. Nevertheless, the cassava linamarin and cyanide were not completely eliminated by the *MeCYP79D1* knockout. Our results indicate that CRISPR/Cas9-mediated mutagenesis is as an alternative approach for development of cassava plants with lowered cyanide content.

## Introduction

Cassava (*Manihot esculenta*, Crantz), a root crop, is predominantly grown in tropical and sub-tropical countries for its carbohydrate-rich roots and is the fourth leading calorie-producing crop worldwide (Bull et al., 2018). Cassava can grow well in minimal rainfall conditions and less fertile soil types; their roots can survive in the soil for 1–2 years without decaying (Tomlinson et al., 2018). These agronomic attributes make cassava a popular food crop, especially for approximately 5 million subsistence farmers in Africa, which is prone to drought and hunger, thereby making it a valuable food security crop (Jarvis et al., 2012). In particular, cassava leaves are consumed in west African countries and are an excellent source of proteins, vitamins, fibers, and minerals (Saurabh et al., 2014). Moreover, both cassava leaves and tubers can be used as animal feed as well as in starch, ethanol, and textile manufacturing industries (Alla et al., 2013).

With the exception of seeds, all cassava tissues contain potentially toxic levels of cyanogenic glycosides, i.e., 95% linamarin and 5% lotaustralin. Cyanogenic glycosides are used by the plant as herbivore repellants (Siritunga and Sayre, 2004) and have recently been found to play a role in the transport of nitrogen from leaves to roots in its reduced form (Islamiyat et al., 2018). In the presence of enzymes, cyanogenic glycosides subsequently undergo hydrolysis to yield hydrogen cyanide (HCN) (Li et al., 2020). Although edible storage roots possess a higher cyanide (CN) level (10–500 mg CN equivalents/kg dry weight) than the maximum recommended levels for foods (10 mg CN equivalents/kg dry weight), this level is approximately 20-fold lower than that in leaves (200–1,300 mg CN equivalents/kg dry weight), which is reportedly the highest CN content (Echeverry-Solarte et al., 2013). Consumption of poorly processed cassava, particularly by a nutritionally compromised population (low protein intake), results into cyanide-related health disorders. The severity of these disorders depends on the level and frequency of cyanogen exposure and the pre-existing health condition of the consumer. Chronic, low-level cyanide exposure has been associated with the development of tropical ataxic neuropathy and hyperthyroidism, whereas acute cyanogen poisoning has been associated with outbreaks of konzo, a rapid and permanent paralysis, which in most cases causes death (Kashala-Abotnes et al., 2019). A contributing factor for cyanide-induced disease is cysteine levels in the diet since sulfur-containing amino acids are required for converting cyanides into less toxic thiocyanates (Blagbrough et al., 2010).

Numerous approaches have been employed to lower the levels of cyanogenic glycosides in cassava. The most frequent traditional procedures used are pounding or tissue-maceration, boiling, sun-drying, baking and steaming, and fermentation (Ani, et al., 2019). Tissue rupture liberates linamarin from the vacuole, allowing linamarase to de-glycosylate it. Boiling is not an efficient method because of the high temperatures involved and only has a 50% cyanogen elimination rate (Taleon et al., 2019). Owing to a processing temperature of ≥100°C and the durability of linamarin in neutral or weak acid conditions, the cyanide elimination rate by baking and steaming remains negligible. In comparison to the other traditional methods, sun-drying is rather effective and is associated with a relatively low cyanide retention rate because a temperature of <55°C is employed, which is the optimal temperature for linamarase activity and cyanogen breakdown (Airaodion et al., 2019). These methods are time-consuming and do not guarantee the complete removal of cyanogenic glycosides, thereby generating harmful food products.

Cassava breeding strategies for reducing the production of cyanogenic glycoside have been explored using conventional technologies. These strategies are targeted at combining pest and disease resistance with favorable agronomic features and minimal cyanogenic potential (Zhang et al., 2017). Because most subsistence farmers choose highly cyanogenic cassava cultivars, these strategies have proved ineffective. The reasons for this preference include taste preference, herbivory reduction, and theft protection (Naveena et al., 2021). Furthermore, cassava crops with a low cyanogenic potential do not produce a high yield of tubers owing to a reduced level of nitrogen transfer from the leaves to the roots, which is a precursor for the production of asparagine, an amino acid present in the roots (Cressey and Reeve, 2019). Due to an extended life cycle, high heterozygosity of allopolyploid plants, poor bloom and seed set, and inbreeding depression as well as the time-consuming and labor-intensive nature of the techniques employed to reduce cyanogenic glycoside levels, conventional cassava breeding does not seem feasible (Ceballos et al., 2004). This has necessitated alternative technologies that would precisely alter the genes responsible for synthesizing cyanogenic glycosides (Behera and Ray, 2017).

Owing to the ability to precisely alter the genomes of living organisms, genome editing (GE) has transformed biological research. GE techniques include zinc finger nucleases (ZFNs), transcriptional activator-like effector nucleases (TALENs), and clustered regularly interspaced short palindromic repeat (CRISPR)/CRISPR-associated nuclease (Cas) system (Kamburova et al., 2017). ZFNs are DNA cleavage proteins with the ability to cleave DNA sequences at any location in the molecule. Although ZFNs and TALENs have been widely employed since 2002 and 2011, respectively, as GE techniques for humans, animals, and plant cells, their effectiveness is limited (Samanta et al., 2016). ZFNs frequently generate unintended off-target mutations owing to their limited specificity. Construction of vectors for ZFNs and TALENs is a time-consuming and effort-intensive process (Juma et al., 2021). Consequently, since 2013, the focus has shifted to the use of CRISPR/Cas9, and more recently, to various novel CRISPR/Cas variants (Liu et al., 2020).

CRISPR/Cas has higher efficiency and success rate, as well as lower cost, than any other GE technique. Several GE techniques, including CRISPR/Cas9, have recently been established in cassava, with applications ranging from yield enhancement to drought tolerance (Odipio et al., 2017), disease resistance (Gomez et al., 2019; Veley et al., 2021), and herbicide tolerance (Hummel et al., 2018).

Production of cyanogen in cassava continually occurs in intact plants through a mechanism involving valine and/or isoleucine as a precursor (Hamza et al., 2020). The process is catalyzed by highly similar proteins, namely CYP79D1/D2 (85% identical). They catalyze the conversion of precursors to their respective oximes (Tawanda et al., 2017). Their production varies naturally among cultivars, indicating that cyanogen levels can be modulated without altering other desirable plant properties. Previous work demonstrated that CYP79D1 is a good target for reduction the levels of cyanogenic glycosides levels in cassava leaves and roots (Siritunga and Sayre, 2003; Jørgensen et al., 2005; Piero, 2013). Here, we focused on the *MeCYP79D1* gene, which encodes the valine monooxygenase I enzyme involved in biosynthesis of cyanogen in cassava. Therefore, we considered the *CYP79D*1 gene a suitable target for generating acyanogenic cassava lines using the CRISPR/Cas9 system. Thus, we aimed to considerably reduce the potentially toxic cyanogenic glycoside levels in cassava plants by knocking out the *CYP79D1* gene.

## Materials and methods

### Plant materials and growth conditions

The cassava plants (TMS 60444) were obtained from International Livestock Research Institute’s *in vitro* germplasm in Nairobi, Kenya, and maintained on a cassava micropropagation medium: Murashige and Skoog (MS) basal salt with vitamins (Murashige and Skoog, 1962) supplemented with 2% sucrose and 3 g/L gelrite at pH 5.8.

### Identification of the cassava cytochrome P450 *(CYP79D1*) gene and CRISPR/Cas9-gRNA vector construction

The reference cassava genome was searched using BLAST for nucleotide sequences that were identical to the *Manihot esculenta* valine monooxygenase I gene in Phytozome 13 (version 8.1) (https://phytozome-next.jgi.doe.gov/; Bredeson et al., 2016). A single copy of the candidate gene, *MeCYP79D1*, was identified. The CRISPOR algorithm (http://crispor.tefor.net/), an online tool, was used to select a gRNA target within the *CYP79D1* gene (**Supplementary Material 1A**) (Concordet and Haeussler, 2018). The chosen gRNA target was located in the exon 3 of the genomic locus LG13 (**Supplementary material 1A**). An off-target analysis was performed for the chosen gRNA target sequence, alongside other considered *CYP79D1* targets, using the Cas-OFFinder online software (http://www.rgenome.net/cas-offinder/) (**Supplementary Material 2**). A pair of complementary DNA oligonucleotides was synthesized (Sigma Aldrich, USA). The oligonucleotides were annealed and the resulting duplex was then cloned into the empty plant CRISPR‐Cas9 vector (Sigma Aldrich) digested by *BsaI*.

The empty plant CRISPR‐Cas9 vector construct included the SpCas9 coding sequence from *S. pyogenes* as well as a gRNA scaffold with a *BsaI* restriction site to facilitate the insertion of a single gRNA. Hygromycin-resistance gene (*hptII*) was also included upstream of the gRNA to aid the selection of transgenic cells during the regeneration cycle. Cas9 expression was driven by the 35S Cauliflower mosaic virus promoter, while gRNA expression was driven by the U6-26 promoter (**Supplementary Material 1B**). PCR and Sanger sequencing were used to confirm the presence of the integrated gRNA and its stability. The binary plasmid, pCRISPR/Cas9-MeCYP79D1 (**Supplementary Material 1B**), carrying the gRNA was transformed into the *Agrobacterium tumefaciens* strain GV3101 using freeze–thaw method.

### *Agrobacterium*-mediated transformation of the cassava cultivar TMS 60444

*Agrobacterium-*mediated transformation was utilized to deliver CRISPR/Cas9 gene editing tools into immature leaf lobes of the cassava cultivar, TMS 60444. One week old emerging leaf lobes from *in vitro* grown cassava plants were harvested and subsequently injured using sterile scalpel blades. Approximately 25 µL of *Agrobacterium tumefaciens* (strain GV3101) bacterial suspension from an infection medium was administered to explant tissues and incubated for 5 min in the dark. The leaf explants were infected with bacterial suspension with an empty vector as a negative control. Using a sterile filter paper, excess infection medium was drained and the explant tissues were placed with adaxial side touching the solid co-cultivation medium (MS basal salts supplemented with 2% (w/v) sucrose, B5 vitamins, 100 mg/L casein hydrolysate, 0.5 mg/L CuSO4, 10 mg/L 2,4-dichlorophenoxyacetic acid (2,4-D) supplemented with 200 µM acetosyringone, 3 g/L gelrite at pH 5.8). The plates containing the explants in co-cultivation medium were wrapped with parafilm to prevent contamination and incubated in the dark for 3 days at 28°C for the incorporation of the *Agrobacterium* into the leaf explants and provide optimal condition for callus formation.

After co-cultivation with *A. tumefaciens* strain GV3101 for 3 days, explants were transferred to a solid resting medium (MS basal salts supplemented with 2% (w/v) sucrose, B5 vitamins, 100 mg/L casein hydrolysate, 0.5 mg/L CuSO_4_, 10 mg/L 2,4-D and 3 g/L gelrite, supplemented with 250 mg/L timentin) for 2 days to inhibit any further growth of *Agrobacterium*, incubated in the dark at 28°C. Copper sulfate was added to increase the growth and visibility of embryo. As a negative control, a different batch of explants co-cultured with *A. tumefaciens* harboring an empty vector were placed in the resting media.

Live calli formed were transferred from the resting medium to a selection medium (MS basal salts supplemented with 2% (w/v) sucrose, B5 vitamins, 100 mg/L casein hydrolysate, 0.5 mg/L CuSO_4_, and 10 mg/L 2,4-D supplemented with hygromycin (10 mg/L), and timentin (15 mg/L) to select for putatively transformed callus, incubated in the dark at 28°C (Syombua et al., 2019). After 2 weeks of culture, the proliferating callus was dissected into smaller pieces to ensure solid contact with the media before being transferred onto a fresh second selection medium supplemented with 20 mg/L hygromycin and 15 mg/L timentin. The callus was incubated in the selection medium for 4 weeks. When the calli were 8 weeks old, they were moved onto the solid MS medium enhanced with B5 vitamins, 2% sucrose, 1 mg/L 1-naphthaleneacetic acid (NAA), 20 mg/L hygromycin, and 15 mg/L timentin, and left in the dark at 28°C; they were cultured for a maximum of 4 months and sub-cultured every 2 weeks. The cotyledon-stage embryos were selected and sub-cultured in a somatic embryo maturation medium, containing MS medium supplemented with B5 vitamins, 2% sucrose, 1 mg/L 6-benzylaminopurine (BAP), 0.01 mg/L NAA, 0.5 mg/L gibberellic acid (GA_3_), 20 mg/L hygromycin, 15 mg/L timentin, 3 g/L gelrite and a pH of 5.8, and placed in the dark at 28°C. The number of cotyledonary embryos generated from each callus was counted after 4 weeks of culturing. For phenolic compound adsorption, green cotyledonary embryos with distinct shoot and root apices were placed in glass bottles containing 50 mL of hormone-free desiccation solution. This medium contained 0.8% activated charcoal, 2% sucrose, MS salts, and B5 vitamins, all of which were solidified with 3 g/L gelrite and incubated at 28°C under 16-h/8-h light/dark cycle.

After 7–14 days, the number of germinated embryos was counted and transferred to a cassava micropropagation medium containing MS salt with vitamins supplemented with 3% sucrose, 5 mg/L hygromycin, 10 mg/L timentin, and solidified with 3 g/L gelrite (pH 5.8); the embryos in this medium were subsequently incubated at 28°C under 16-h/8-h light/dark cycle. Putative gene-edited cassava plants with distinct shoots and roots were carefully removed from the medium to minimize root damage and transferred to small pots filled with peat moss and covered with a plastic bag; these pots were placed in a glasshouse for 2 weeks at room temperature to regulate humidity and temperature. Using a hand sprayer, a mist of water (50 mL) was added. After 2 weeks, the plastic bags were gradually removed to allow the plantlets to acclimatize to the glasshouse environment. The surviving plants were then transplanted to larger pots filled with a mixture of peat moss and forest soil (50/50 v/v), and the plants were cultivated on an open bench in a greenhouse maintained at 28°C under natural light and artificial illumination with an approximate light/dark cycle of 16 h/8 h. Subsequently, the plants were placed into potted soil, and the watering interval (150 mL) was reduced to once a week.

### PCR-based selection of *mecyp79d1* mutants and characterization of mutagenesis events

Leaf tissues were collected from 3 months old acclimatized *in vitro* regenerated plants. Approximately 0.5 g of tissue was placed in a 2-mL Eppendorf tube containing ceramic beads and vortexed to fine powder. Genomic DNA was extracted using the cetyltrimethylammonium bromide (CTAB) method (Doyle and Doyle, 1990) and treated with RNase A to remove RNA contamination. The putative transgenic plants were subjected to PCR analyses to confirm the integration of T-DNA using primers specific to the *Cas9* gene and target gene (**Supplementary Material 3**). The *Cas9* gene was amplified using gene-specific primers complementary to a 900-bp amplicon to confirm the presence of the *Cas9* gene. Each PCR reaction was performed in a 20 µL (total volume) of reaction mixture. The Cas9 gene was amplified under the following conditions: 95°C for 5-min initialization; 35 cycles of denaturation at 95°C for 10 s, annealing at 53°C for 30 s, extension at 72°C for 1 min; and a final extension of 72°C for 5 min. Amplification with gene-specific primers (*CYP79D1*; 350 bp in length) confirmed the gene of interest. The following conditions were used to perform the PCR reaction: initialization at 95°C for 5 min, denaturation at 95°C for 10 s, annealing at 53.83°C for 30 s, extension at 72°C for 1 min, and a final extension at 72°C for 5 min. A negative control containing 2 µL of non-transformed plant DNA and a positive control containing 2 µL of plasmid DNA were run alongside the DNA extracted from putatively transformed plants. PCR products were resolved on a 1.5% gel and visualized under UV light. Amplicons from target gene-specific primers were washed using Exo SAP-IT (Thermo Fischer Scientific, USA) and subjected to targeted Sanger sequencing using CYP79D1 F and CYP79D1 R primers to characterize CRISPR/Cas9-induced mutations. Raw sequences were trimmed, with overlapping paired-end reads were merged into a single sequence and aligned with the wild-type reference sequence of the *MeCYP79D1* gene using Bioedit alignment software v7.2 with default settings to identify any insertions or deletions (indels). Genome editing efficiency was calculated by dividing the total number of transgenic lines by the number of mutant lines.

### Analysis of *Cas9* gene expression in mutants

Total RNA was extracted from the leaf tissues (30 mg) of both *in vitro* putative transgenic plants and non-transgenic control cassava plants using RNeasy Mini Kit (Qiagen, GmbH, Hilden, Germany) according to the manufacturer’s instruction, and On-Column DNase digestion was performed for 15 min at room temperature using 1 unit DNase (Invitrogen, Carlsbad, CA). RNA concentration was measured using Nanodrop ND-2000 spectrophotometer. DNase was inactivated according to the manufacturer’s instructions to avoid the digestion of newly synthesized cDNA. First strand cDNA synthesis was performed using 1 µg of total RNA and reverse transcriptase from the LunaScript™ RT SuperMix Kit according to the manufacturer’s protocol. The synthesized cDNA was amplified by PCR using 1× PCR buffer, 1.5 mM MgCl_2_, 0.1 mM dNTP, 2.5 units of *Taq polymerase*, 0.4 μM of each primer specific for *Cas9* gene primer sequences (**Supplementary Material 3**). To check the quality of the synthesized cDNA, Actin-7 (ACT) amplification was performed as an internal control using ACT specific primers (**Supplementary Material 3**). The RT-PCR products were subsequently run on an agarose gel [1% (w/v)] at 100 V for 45 min and visualized under UV light.

### Measurement of cyanogenic potential

#### Measurement of linamarin from *in vitro* plantlets

High performance liquid chromatography (HPLC) was used to measure linamarin content in plantlets grown *in vitro*. Approximately 5 g of 4 months old cassava leaves were harvested and immediately homogenized in a blender for 14 s at low speed, followed by homogenization for 1 min (2×) at high speed with 25 mL of 0.25 M chilled sulfuric acid. To remove insoluble materials, the homogenates were filtered using a filter cloth. The homogenizer jar was washed with 40 mL of the 0.25 M chilled sulfuric acid before filtering the homogenates again in the aforementioned manner. Negative control was a chilled 0.25 M sulfuric acid solution without any plant tissues. The filtrates were centrifuged for 10 min at 10,000 rpm at 4°C. The clear supernatant fluid was collected and stored at −20°C. To facilitate absolute quantification of linamarin, a standard stock was prepared from solid linamarin (A.G. Scientific, Biochemical Manufacturer, USA; purity ≥ 98) resuspended in water to obtain a stock solution of 100 µg/mL and stored at −20°C. While performing the assay, the standard was serially diluted at concentrations of 10–100 µg/mL (10, 20, 40, 60, 80, and 100) and subjected to HPLC analysis. Three technical replicates of the standard stock were prepared.

Cassava extract samples were analyzed using a HPLC system, with the mobile phase consisting of methanol and water (25:75, v/v). A C18 HPLC column was used for the analysis, with a flow rate of 1 mL/min and an injection volume of 10 µL (column temperature 40°C). Detection was done at a wavelength of 214 nm. The gas bubbles from the mobile phase were removed before use. HPLC data acquisition and analysis were performed using an Excel spreadsheet. The fresh weight of linamarin in the plant extracts was measured by plotting a calibration curve of the peak area values obtained from the serially diluted linamarin standard solution and by using the following formula: y=mx + c; here, *y* was the concentration of crude linamarin, and *x* was the peak area reading obtained from HPLC.

#### Measurement of cyanide from acclimatized plantlets grown *in vitro*

The total cyanide concentration in the leaves of hygromycin-resistant transgenic TMS 60444 cassava lines and wild-type cassava carrying the empty vector was measured using a picrate assay kit (Bradbury et al., 1999). Young cassava leaves were harvested from 4 months old acclimatized cassava, chopped using scissors, and ground immediately. The buffer discs were placed in a flat-bottomed bottle, and 100 mg of the ground leaves were placed on top of it. Clean water (1.0 mL) was added using a plastic pipette and the solution was mixed gently. A yellow indicator paper was immediately added without allowing it to touch the liquid, and the bottle was immediately closed with the screw-capped lids. This process was performed in triplicate (biological replicates) for each sample. For the positive control, the buffer/enzyme paper disc was placed in a bottle along with the standard pink paper disc, with the subsequent addition of 1 mL of clean water. A yellow indicator paper was then placed and the bottle was closed tightly using the screw-capped lid. For the negative control, the standard pink paper was placed in a bottle with 1 mL of water added to it, and the bottle was closed tightly using the screw-capped lid. All bottles were subsequently incubated at room temperature for 24 h.

The bottles were opened, and the indicator papers matched against the color shades on the color chart provided in the cassava cyanogen kit. The total cyanide content of the leaves was estimated in parts per million (ppm), equivalent to HCN (mg)/cassava fresh weight (kg), based on the readings given in the color chart, with the negative control being zero and the positive control producing a color equivalent to an estimated cyanide concentration given in ppm. The plastic backing of the indicator paper was carefully removed and the paper was placed in a test tube followed by the addition of distilled water, accurately measured to 5.0 mL. The test tubes were incubated at room temperature for about 30 min, with intermittent gentle stirring. The absorbance of the solutions was measured at 510 nm, and the value of the negative control was subtracted subsequently. The total cyanide content in ppm was calculated using the equation: total cyanide content (ppm) = 396 × absorbance.

### Agro-morphological characterization of gene-edited mutants

Wild-type and transgenic TMS 60444 cassava plantlets were grown in the greenhouse under normal conditions. The agro-morphological traits were characterized by measuring plant height, leaf length, leaf width, number of leaves per plant, and stalk length after being incubated for 4 months in the green house (Nadjiam et al., 2016).

### Statistical analysis

A statistical analysis of the phenotypic data and expression levels was performed by employing two-tailed Student’s t-test in the GraphPad Prism software (*p<0.05).

## Results

### Cas9/gRNA *Agrobacterium*-mediated transformation

**Figure 1:**
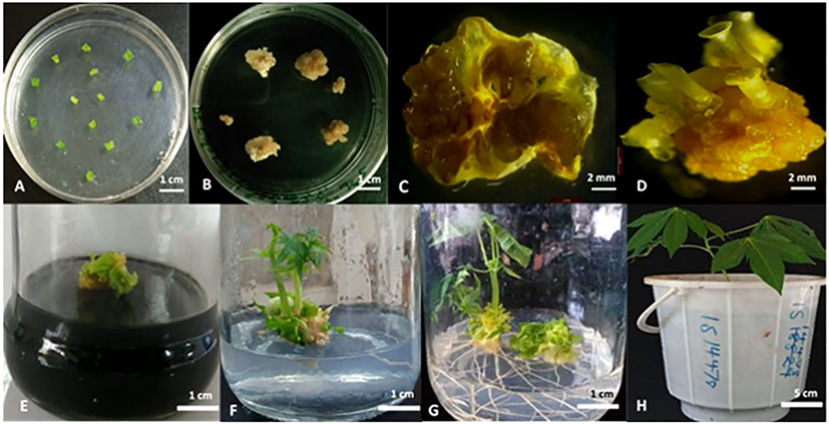
Cas9/gRNA *Agrobacterium*-mediated cassava transformation and recovery of transgenic cassava plants. **(A)** Co-cultivation of immature leaf lobes with *Agrobacterium* harboring the DNA construct; **(B)** *Agrobacterium*-infected explants in callus induction media under selection; **(C)** Non-transformed leaf explant in selection medium; **(D)** Mature callus with the cotyledonary embryos; **(E)** Desiccation of shoots in activated charcoal (0.8%); **(F)** Rooting in hormone free MS media; **(G)** Plantlets after 12 days in rooting media; **(H)** Putatively transformed cassava hardened in glasshouse. Scale: 1 cm **(A, B, E-G)**, 2 mm **(C, D)**, and 5 cm **(H)**.

To generate transgenic plants with targeted mutations in *MeCYP79D1* gene, a gene-specific CRISPR/Cas9 vector construct was designed and used for transformation. A ranked list of prospective off-target sites was produced using the Cas-OFFinder online tool (http://www.rgenome.net/cas-offinder/), which was utilized to evaluate for potential off-targets (**Supplementary Material 2**). This sorted list of probable off-target sites aids in the selection and assessment of intended target sites, assisting in the development of CRISPR/Cas systems with low off-target effects, as well as the identification and measurement of CRISPR/Cas induced off-target cleavage in cells. The gRNA for CRISPR/Cas9 editing was synthesized using the target sequence highlighted in yellow (**Supplementary Material 2**). *Agrobacterium*-mediated transformation and somatic embryogenesis were performed as previously described by Odipio et al. (2017) and Syombua et al. (2021). After repeatedly subculturing the tissue and performing antibiotic selection on a selective medium (**Figures 1A–C**), multiple hygromycin-resistant cotyledon-stage embryos were generated from original leaf explants (**Figures 1D, E**). Culturing of antibiotic-resistant continued on a hygromycin-containing medium, consequently producing plantlets on a selective medium (**Figures 1F, G**). In total, 5.91% independent lines of the regenerating somatic embryos were recovered after selection on the hygromycin-containing-medium (**Supplementary Material 4**); 1.78% of the leaf explants germinated to produce plantlets, thereby generating eight independent transgenic plant lines which subsequently propagated in the greenhouse (**Supplementary Material 5**).

### Molecular analysis of CRISPR/Cas9-induced mutations in the *MeCYP79D1* gene

To validate exogenous T-DNA insertion in the transgenic plantlets, DNA extracted from eight independent transgenic events were analyzed by PCR to detect the presence of T-DNA and to amplify the targeted *MeCYP79D1* gene using gene-specific primers for the Cas9 and target gene (**Supplementary Material 3**). All the putative transgenic plants tested positive for the presence of Cas9, which were referred to as TMS1, TMS2, TMS3, TMS4, TMS5, TMS6, TMS7, and TMS8, respectively; however, no amplification was observed for the wild-type (**Supplementary Material 6A**). A 350-bp fragment corresponding to the target region of the *MeCYP79D1* gene in cassava was amplified in all putative transgenic cassava plantlets as well as in the wild-type cassava plantlet (**Supplementary Material 6B**).

### Detection of targeted mutation in putative transgenic cassava

**Figure 2:**
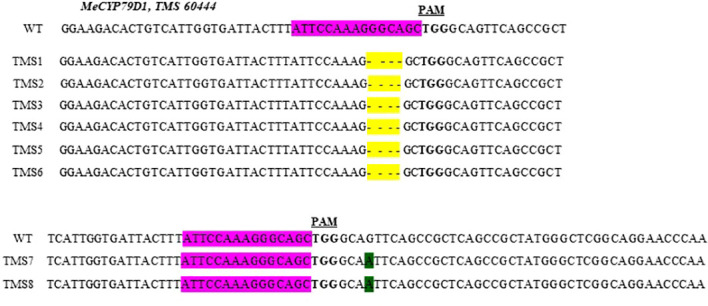
Partial sequences of the *mecyp79d* mutant alleles aligned with respective wild-type (WT) sequences. Sequences from each mutant plant (TMS1 - TMS8) are shown below the respective WT reference sequences. Nucleotide deletions are highlighted in yellow, while substitutions – in green. The *MeCYP79D1* target region is highlighted purple in the wild-type (WT) reference sequence, with the protospacer adjacent motif (PAM) shown in bold font.

The nature of mutation acquired in putative transgenic cassava plantlets was evaluated by performing targeted Sanger sequencing of PCR amplicons. Mutations in the *MeCYP79D1* gene were observed in all transgenic plants (**Supplementary Material 7**). Both nucleotide deletions and substitutions were observed (**Figure 2**). According to the mutations detected, the mutagenesis efficiency of pCRISPR/Cas9–MeCYP79D1 construct was 100% (8/8). Deletions occurred more frequently than substitutions (**Figure 2**). Combined analysis of the results indicated that deletions were the most frequent type of mutation (75%), with four base-pair deletions, which occurred three nucleotides upstream of the PAM sequence, and found in the following T0 plants: TMS1, TMS2, TMS3, TMS4, TMS5, and TMS6. These deletions occurred within the target site of the gene of interest. Meanwhile, substitution was observed in the TMS7 and TMS8 samples, accounting for 25%, with one nucleotide substitution (substitution of nucleotide “G” with “A”) reported. The substitutions were observed four nucleotides downstream of the PAM site, which was located outside the target site (**Figure 2**). The sequence analysis of the wild-type TMS 60444 amplicons showed no INDELs as well as substitutions in the target region of the gene of interest (**Figure 2**). Thus, the results suggested that pCRISPR/Cas9–MeCYP79D1-gRNA effectively and precisely guided Cas9-mediated genomic DNA cleavage.

### RT-PCR analysis of putative transgenic plantlets

RT-PCR analysis detected *Cas9* gene expression in all eight transgenic lines of the cultivar TMS 60444 but not in the wild-type line (**Supplementary Material 8**), showing *Cas9* gene expression in the transgenic cassava plants. PCR amplification products of *Cas9* measuring 900 bp in length were identified in the transformants, which was consistent with the expected size of amplicons. However, no Cas9 PCR amplicons were observed in the wild-type control (**Supplementary Material 8A**). *Actin7* primers successfully amplified the target sequence in the case of cDNA derived from wild-type plants (**Supplementary Material 8B**).

### Cyanogenic potential of gene-edited plants

#### HPLC measurement of linamarin from *in vitro* plantlets

**Figure 3:**
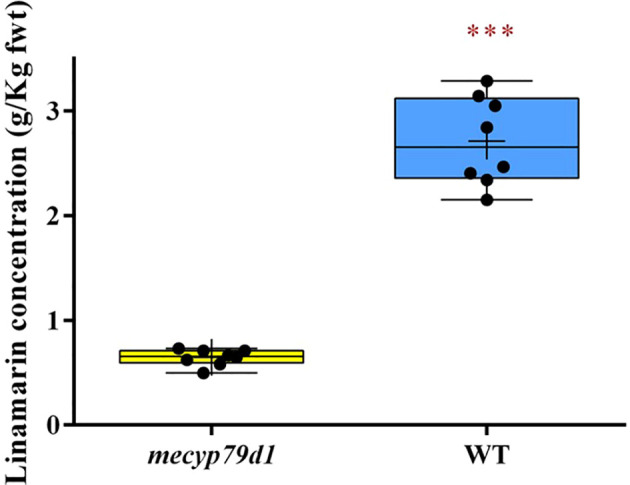
Total linamarin concentration in crude extract from freshly ground cassava leaves. Asterisk indicates statistically significant difference at p<0.05). fwt (fresh weight).

Linamarin levels in the leaves of gene-edited TMS 60444 cassava plantlets were measured using HPLC, with age-matched wild-type cassava plantlets being positive controls. The HPLC readings of the linamarin standard provided the peak areas, which were used to generate the linear response graph curve. Linamarin concentration in the crude extract of non-transgenic (WT) plantlets ranged from 2.405 to 3.143 g/kg fresh weight, whereas that in transgenic (*mecyp79d1*) plantlets ranged from 0.496 to 0.731 g/kg fresh weight (**Figure 3**). The results revealed high linamarin content in non-transgenic (WT) cassava plantlets with a mean fresh weight of 2.72 ± 0.17 g/kg in comparison with 0.65 ± 0.03 g/kg in transgenic (*mecyp79d1*) cassava plantlets (**Supplementary Material 9**).

##### Cyanide measurement from *in vitro* plantlets

**Figure 4:**
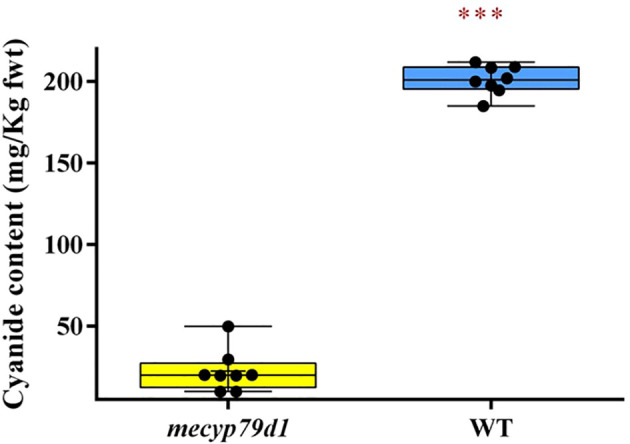
Total cyanide content in fresh cassava leaves. Asterisk indicates statistically significant difference at p<0.05). fwt (fresh weight).

Besides linamarin quantification, cyanide levels were also measured in the leaves of wild-type and mutant TMS 60444 acclimatized plantlets using picrate assay (Bradbury et al., 1999) (**Figure 4**). Because the cyanide content can vary considerably between the leaves of the same plant and among different plants of the same cultivar (Siritunga and Sayre, 2004), three different leaf samples were analyzed per mutant plant to account for experimental variability. The mean cyanide level in non-transgenic (WT) plants was 201.0 ± 0.99 mg/kg fresh weight, which was considerably higher than that in transgenic (*mecyp79d1*) plants at 17.44 ± 2.482 mg/kg fresh weight (**Supplementary Material 10**). Therefore, MeCYP79D1 gene knockout in cassava led to a drastic reduction in cyanide content relative to their wild-type counterparts (**Figure 4**).

### Characterization of agronomic traits

**Figure 5:**
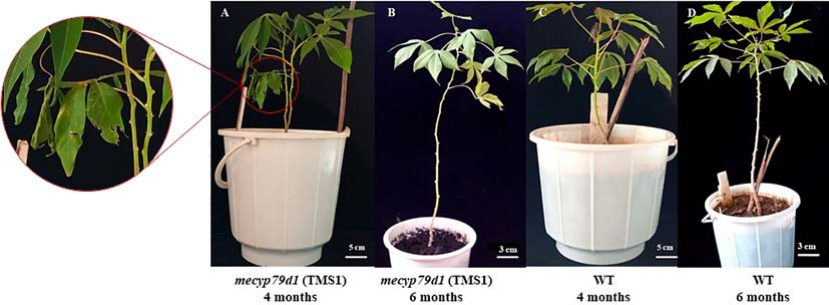
Morphological phenotype of the TMS1 mecyp79d grown alongside the wild-type (WT) control in the greenhouse. **(A)** 4 month old TMS1 mecyp79d cassava plant; **(B)** 6 month old TMS1 mecyp79d cassava plant; **(C)** 4 month old WT cassava plant; **(D)** 6 month old WT cassava plant.

**Figure 6:**
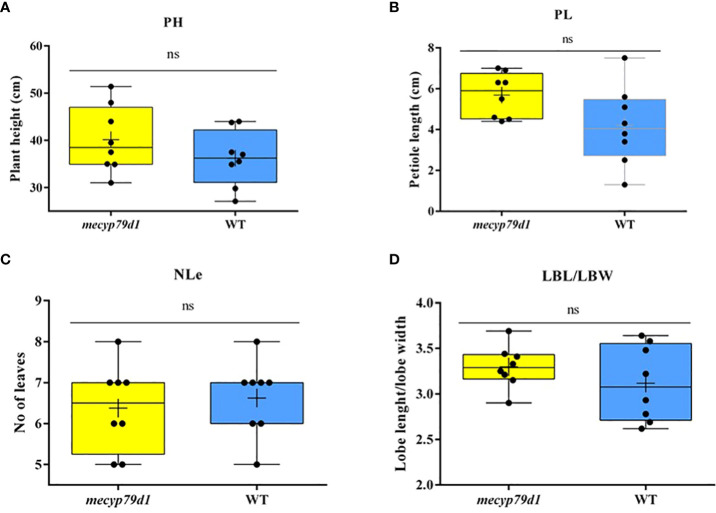
Phenotyping for agronomic traits of 4 month old *mecyp79d* and wild-type (WT) cassava plants. **(A)** Plant height; **(B)** Petiole length; **(C)** The number of leaves per plant; **(D)** The ratio between the leaf lobe length and the width. PH (plant height); petiole length (PL); NLe (number of leaves); LBL (lobe length); LBW (lobe width); ns (not significant). “ns” indicates the values are not significantly different (p>0.05).

Mutant and wild-type TMS 60444 cassava plants were analyzed for plant height, leaf length, leaf breadth, petiole length, and the number of leaves per plant to confirm if mutations in the *MeCYP79D1* gene alter agronomic parameters (**Figure 5**). No significant difference was noted between transgenic and non-transgenic cassava plants for any of the evaluated agronomic traits (**Figure 6**; **Supplementary Material 11**); however, the transgenic plant lines suffered from insect infestation and their leaves were destroyed between 4 and 6 months of storage in the greenhouse (**Figures 5A, C**). After spraying the transgenic plants with insecticides, they were seen to grow well but had thinner stems than the non-transgenic lines (**Figures 5B, D**).

## Discussion

Functional genomics as well as molecular design and breeding studies of cassava fundamentally revolve around the scope to modify its genome to create mutant plant lines. Recently, CRISPR/Cas9 gene editing technology has substantially evolved, allowing scientists to edit and knock out plant target genes as well as create mutants in a convenient, quick, and efficient manner (Chen et al., 2019). Despite the emergence of CRISPR/Cas9 genome editing technology, to date, only few reports are available on its use for trait improvement and biotic stress resistance in tropical cassava (Juma et al., 2021). Cassava genome editing has the potential to create new avenues for resolving biotic and abiotic restrictions in cassava production and post-harvest utilization (Odipio et al., 2017).

We therefore exploited the CRISPR/Cas9 system for targeting the cassava *CYP79D1* gene which encodes a key enzyme in the cyanogenic glycoside biosynthesisfor key enzyme in the cyanogenic glycoside biosynthesis (Siritunga and Sayre, 2003). Silencing the *CYP79D1* by RNAi resulted in lowered cyanogenic glycosides in cassava due to impaired biosynthesis of cyanogenic glycosides (Liu et al., 2017). To effectively knockout the *CYP79D1* gene in cassava using the CRISPR/Cas9 system, a single guide RNA was designed for the *CYP79D1* genomic region. Existing genetic transformation systems (Odipio et al., 2017; Syombua et al., 2019) were employed to integrate the CRISPR/Cas9 tools into the embryogenic cells. Through *A. tumefaciens*-mediated cassava transformation and antibiotic selection as the primary methods used for delivering CRISPR/Cas9 components, eight independent positive transgenic plantlets were obtained (**Figure 1H**). This is because transfection with *Agrobacterium* typically results in simple gene insertion events of transfer DNA (T-DNA) sequence in a binary plasmid, with a low frequency of transgene silencing (Wang et al., 2016). The *A. tumefaciens* strain GV3101 was utilized in the study to deliver the pCRISPR/Cas9–MeCYP79D1 construct into leaf explants because it has previously been shown to have a high transformation rate of up to 65% (Chetty et al., 2013). The study describes a rapid, simple, effective, and stable *Agrobacteriu*m-mediated strategy for transforming TMS 60444 plant cultivars with *A. tumefaciens* strain GV3101 carrying the pCRISPR/Cas9– MeCYP79D1plasmid containing the *hptII* selectable marker to facilitate selection of transgenic cassava plants using hygromycin antibiotic.

In the present study, cassava regeneration frequency and transformation efficiency were low, in the range of 5.91-6.82% and 1.33-2%, respectively (**Supplementary Materials 4 and 5**), respectively. Syombua et al. (2021) also reported a similar transformation frequency of 0.5% for TMS 60444 cultivar. The low efficiency observed could be explained by the selective stress of calli caused by hygromycin and timentin antibiotics. Accordingly, hygromycin and timentin had also been used to select transformed tissues in the present study, and both these antibiotics are exceedingly toxic to cassava tissues. Furthermore, the low regeneration frequencies and efficiencies can also be a result of recalcitrant embryo either in the globular stage or during the transition from torpedo to cotyledonary stage (Ochatt and Revilla, 2016).

Molecular analysis was performed on the independent transgenic cassava lines. The findings of PCR and RT-PCR analyses of the putative transgenic lines indicated that the T-DNA derived from pCRISPR/Cas9–MeCYP79D1 construct was stably integrated into the cassava genome. An RT-PCR assay performed on the eight transgenic lines revealed *Cas9* gene expression in all of them. The results revealed that the transformation approach used in this study has high replication potential with regard to genetic transformation of recalcitrant cassava genotypes. This gene editing process for knocking out the *CYP79D1* gene in cassava using CRISPR/Cas9 is consistent with previously reported knockout process followed for other cassava plant genes, including the ones encoding phytoene desaturase (Odipio et al., 2017), the viral AC2 protein (Mehta et al., 2019), protein targeting to starch 1 (PTST-1) and granule-bound starch synthase (GBSS) (Bull et al., 2018), 5-enolpyruvylshikimate-3-phosphate synthase (EPSPS) (Hummel et al., 2018), multiple TFL 1-like floral repressor (Odipio et al., 2018), eukaryotic translation initiation factor 4E (elF4E) isoforms (Gomez et al., 2018), methylesterase 3 (MeSIII) (Li et al., 2020), and MeSWEET10a (Veley et al., 2021).

The nature of mutations within the *CYP79D1* gene among the regenerated T0 plants was identified by Sanger sequencing performed on each individual plant. The sequencing results showed the presence of either indels or substitutions in the CYP79D1 gene in of all of the eight regenerated T0 plant (100%). The indels detected in the TMS1-6 T0 plants were identical 4 bp-long deletions located 3 bp upstream from the PAM site.The deletions reported here were almost certainly caused by nonhomologous end-joining (NHEJ) DNA repair following cleavage by Cas9, as previously reported in cassava subjected to CRISPR/Cas9-mediated alteration of the *PTST-1* and *GBSS* genes (Bull et al., 2018), *MeSIII* gene (Li et al., 2020), and genes encoding elF4E (Gomez et al., 2018). The presence of a single nucleotide mutation downstream of the targeted region might suggest that the homology-directed repair (HDR) pathway was activated to repair the double-stranded breaks (DSBs) occurring in the *CYP79D*1 gene. This rare occurrence seems to indicate that CRISPR/Cas9 can direct mutation outside the target region (Odipio et al., 2017).

Linamarin levels in transgenic plants were significantly lower than those in their wild-type counterparts (*p<0.05*), which were below 10 mg/kg, the set limit for cassava to be classified as safe for consumption. The wild-type cassava showed a higher linamarin level than that established by the Food and Agriculture Organization/World Health Organization as the accepted levell (FAO/WHO, 1999). Complete elimination of the cyanogenic glycoside linamarin, in the genome-edited plants was not achieved because only one gene, namely *CYP79D1*, encoded for the enzyme necessary for catalysis in the first step (dedicated stage) of linamarin production in cassava (Gunasekera et al., 2018). Cassava is a paleotetraploid plant with two genes encoding the CYP79D enzyme. The first step in the biosynthesis of cyanogenic glycosides in cassava is mediated by two enzymes, valine monooxygenase I and valine monooxygenase II, which are encoded by the genes *CYP79D1* and *CYP79D2*, respectively (Bredeson et al., 2016). These two genes are 85% identical. The results were consistent with those of previous studies which used RNAi technology to produce alterations in the functioning of the *CYP79D1* gene in cassava plants, resulting in approximately three-fold lower cyanide concentrations than the cyanide content found in wild-type cassava (Siritunga and Sayre, 2003; Jørgensen et al., 2005; Piero, 2013). CRISPR/Cas9-mediated *CYP79D1* gene modification in *P. pastoris* also resulted in similar aberrations, as reported by Jiang et al. (2021). According to the findings of the present study, CRISPR/Cas9 genome editing technology is more efficient than the RNAi technology, when it comes to reverse genetics as it allows one to completely inactivate the gene by e.g. introducing frame-shift mutations. The CRISPR/Cas9 approach allows for a more precise and faster method of generating gene knockouts in cassava, as compared to traditional breeding methods, without a major impact on agronomic traits in the case of the *CYP79D1* gene. Furthermore, determination of cyanide content using picrate test yielded comparable results to the determination of linamarin concentration. The transgenic plants had a cyanide level which was nearly seven-times lower than that in their wild-type counterparts, which corroborates with the results previously reported by Taylor et al. (2012) and Piero (2013). It was previously reported that CYP79D1/D2 gene knockdown performed using the RNAi technology lowers the cyanide content in cassava leaves and roots by three-fold (Siritunga and Sayre, 2003; Jørgensen et al., 2005; Piero, 2013). The CRISPR/Cas GE technology is therefore superior owing to its efficiency, simplicity, and preciseness.

Analysis of agronomic traits of the transgenic cassava plantlets acclimatized in the greenhouse revealed no substantial difference from those of wild-type plants (**Figure 5**); these were similar to the findings reported by Veley et al. (2021). Nevertheless, some differences were still observed between the transgenic and non-transgenic plants, which could have been caused by either environmental shock or herbivore predation. Herbivores, whiteflies, and aphids attacked the leaves and roots of transgenic plants, while the non-transgenic plants remained unaffected (**Figure 5A**) (Brandt et al., 2020). This could be related to an alteration in the linamarin biosynthesis pathway, which facilitates the defense mechanism of plants by generating hydrogen cyanide when cyanogenic glycosides come in contact with their corresponding enzymes during a herbivore attack. This is similar to the findings of a study conducted by Siritunga and Sayre (2003), who reported that cyanogenic glycosides act as insect repellants in cassava. Furthermore, transgenic plants developed thinner stems than non-transgenic plants likely owing to changes in the biosynthesis of cyanogenic glycosides (**Figure 5B**) (Tawanda et al., 2017). Linamarin is used in the biosynthesis of aspartate, an amino acid, which is subsequently transported to the roots of an intact plant as a translocable form of reduced nitrogen. This promotes the development of big roots and stems (Echeverry-Solarte et al., 2013; Tawanda et al., 2017). Thus, *mecyp79d1*-mutant plants were successfully created in our gene editing study, which also provided deeper insights into the regulatory mechanism of cyanogen synthesis in cassava plants.

## Conclusion

In the present study, we investigated the potential roles of the *CYP79D1* gene in biosynthesis of cyanogenic glycosides in cassava using CRISPR/Cas9 genome editing system. The *mecyp79d1* cassava plants exhibited significant reduction in the levels of cyanogenic glycosides as compared to the wild-type, while showing increased susceptibility to insect pests and grew with thinner stems as compared to the wild-type counterparts. Our study contributes to a better understanding of the underlying role of the *CYP79D1* gene in the biosynthesis of cyanogenic glycosides and downstream importance of the secondary metabolites. In addition, it provides the evidence for feasibility of using CRISPR/Cas9 to precisely edit the *CYP79D1* gene and to determine its function. Thus, our study can pave a way for the editing of other CYP79 genes in cassava, thereby advancing plant biology and helping in solving food security related issues.

## Data availability statement

The original contributions presented in the study are included in the article/**Supplementary Material**. Further inquiries can be directed to the corresponding author.

## Author contributions

BJ performed all the experiments, analyzed the data and wrote the manuscript, AM assisted in some of the investigations and data analysis, CM, MN and WM supervised the work, contributed with experimental design and coordination, and reviewed the manuscript, WM conceptualized the idea. All authors contributed to the article and approved the submitted version.

## Funding

This research was supported by The World Academy of Sciences (Grant No. 18-175 RG/810/AF/AC G - FR3240303653) and the International Centre for Genetic Engineering and Biotechnology (Contract No. CRP/KEN20-03).

## Acknowledgments

We are grateful to Kenyatta University providing the laboratory space at the Plant Transformation Laboratory to perform this work. The authors acknowledge The World Academy of Sciences (TWAS) and the International Centre for Genetic Engineering and Biotechnology (ICGEB) for providing research grants.

## Conflict of interest

The authors declare that the research was conducted in the absence of any commercial or financial relationships that could be construed as a potential conflict of interest.

## Publisher's note

All claims expressed in this article are solely those of the authors and do not necessarily represent those of their affiliated organizations, or those of the publisher, the editors and the reviewers. Any product that may be evaluated in this article, or claim that may be made by its manufacturer, is not guaranteed or endorsed by the publisher.
