# Supplementary material for: Targeted mutagenesis of the CYP79D1 gene via CRISPR/Cas9-mediated genome editing results in lower levels of cyanide in cassava
Source: Front Plant Sci. 2022 Oct 26;13:1009860. doi: 10.3389/fpls.2022.1009860 (PMC9644188; doi:10.3389/fpls.2022.1009860)
Supplement: Supplementary file 1 [file DataSheet_1.docx]

**Supplementary Material**


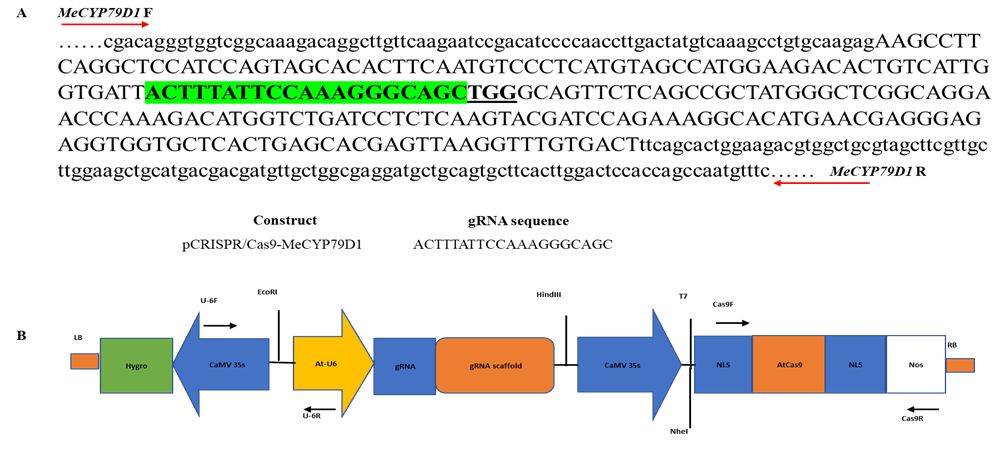


**Supplementary material 1:** Schematic representation of the cassava *MeCYP79D1* target gene, location of the gRNA and the CRISPR/Cas9 gene-editing construct. A: Schematic representation of target region showing the sequences and location of the 20 bp gRNA. gRNA is highlighted in green. Positions of forward (F) and reverse (R) primers flanking the target region in *MeCYP79D1* are indicated with red arrows, respectively. B: The schematic of CRISPR/Cas9 binary vector, pCRISPR/Cas9-MeCYP79D1 used for stable Agrobacterium-mediated transformation of cassava. The gRNA used to target *MeCYP79D1* is driven by the Arabidopsis thaliana promoter (AtU6-26), with the gRNA ligated at the position indicated by the blue box and aided by the HindIII restriction site. The Cauliflower mosaic virus promoter (CaMV 35S) drives Cas9 gene expression, which, in conjunction with inserted gRNA, causes mutations in the target region of the *MeCYP79D1* gene; NLS, nuclear localization signal; Nos, Nos terminator; LB, left border; RB, right border. The positions of forward (F) and reverse (R) primers used to amplify respective cassette regions are indicated by black arrows.

**Supplementary material 2:** Off-target analysis performed using the Cas-OFFinder online tool. The PAM motif for the selected gRNA target (highlighted in yellow) is shown in bold font.

| **Target Sequence** | **Bulge Type** | **Bulge Size** | **Mismatch** | **Number of Off-targets** |
| --- | --- | --- | --- | --- |
| ACTTTATTCCAAAGGGCACC**NGG** | DNA | 1 | 1 | 2 |
| ACTTTATTCCAAAGGGCACCNGG | DNA | 1 | 2 | 8 |
| ACTTTATTCCAAAGGGCACCNGG | DNA | 2 | 2 | 2 |
| ACTTTATTCCAAAGGGCACCNGG | RNA | 1 | 2 | 2 |
| ACTTTATTCCAAAGGGCACCNGG | RNA | 2 | 2 | 10 |
| ACTTTATTCCAAAGGGCACCNGG | X | 0 | 1 | 2 |
| ACTTTATTCCAAAGGGCACCNGG | X | 0 | 2 | 2 |

*
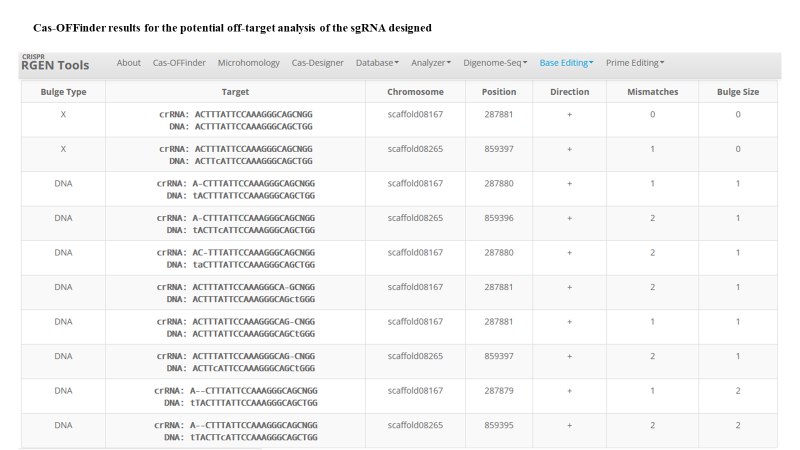
*

**Supplementary material 3:** Primers used to confirm the integration of T-DNA and integrity of CYP79D1target sequence.

| Primer | Forward sequence | Reverse sequence | Product size (bp) |
| --- | --- | --- | --- |
| CYP79D1 | ACCAGGGCCTGAAGAAATCG | CCCAGCTGCCCTTTGGAATA | 350 |
| Cas9 | TGCAGACCTACAACCAGCTG | CCGTTCTTGGACTGGTCGAA | 900 |
| Actin7 | TGCAATGTATGTTGCCATCCAGGC | TTACACCGTCACCAGAATCCAGCA | 300 |

**Supplementary material 4:** Transformation, callus recovery and regeneration of TMS 60444 cassava

| **Experimental batch** | **No of explants** | **Live calli in selection media** | **Calli in germination media** | **Regenerated Shoots** | **Regeneration frequency (%)** |
| --- | --- | --- | --- | --- | --- |
| Negative control | 150 | 0 | 0 | 0 | 0 |
| 1 | 150 | 50 | 44 | 3 | 6.82 |
| 2 | 150 | 67 | 51 | 2 | 3.92 |
| 3 | 150 | 62 | 43 | 3 | 6.98 |
| Average | 150 |  |  |  | 5.91 |

**Supplementary material 5:** Cassava transformation efficiency of TMS 60444

| Experiment batch | No. of explants used | No. of Transgenic plants | No. of PCR positive | Transformation efficiency (%) |
| --- | --- | --- | --- | --- |
| Negative control | 150 | 0 | 0 | 0 |
| 1 | 150 | 3 | 3 | 2.00 |
| 2 | 150 | 2 | 2 | 1.33 |
| 3 | 150 | 3 | 3 | 2.00 |
| **Total** | 450 | 8 | 8 | 1.78 |


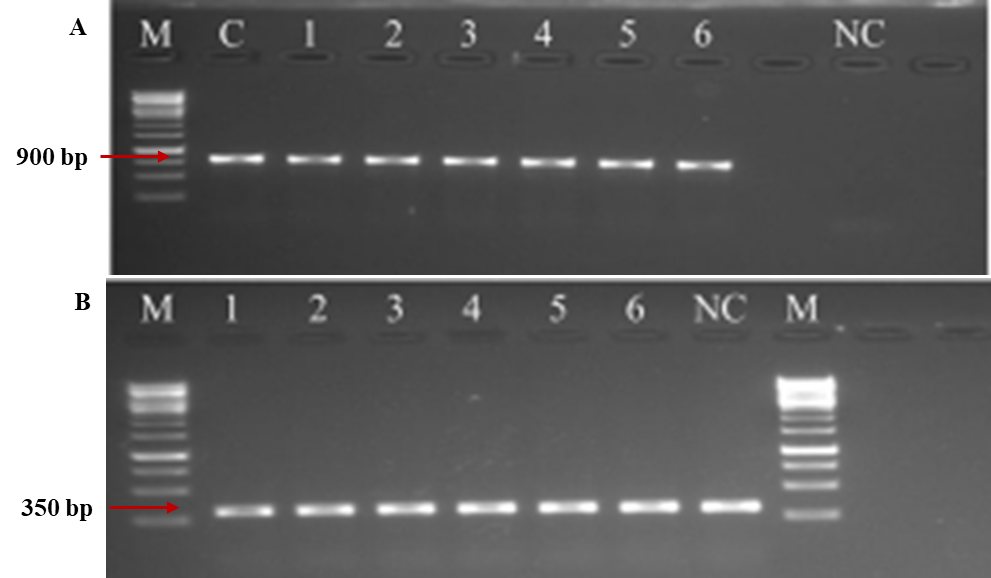


**Supplementary material 6:** PCR amplification and detection of CRISPR/Cas9 T-DNA integration and the *MeCYP79D1* target sequence. A: Confirmation of CRISPR/Cas9 presence by detection of the Cas9 gene. B: PCR amplification for 350 bp of the *MeCYP79D1* target region. The expected band sizes are as shown on the left as 900 and 350 bp for (A - B) respectively. M is molecular marker. For (A) C is plasmid DNA containing the binary vector pCRISPR/Cas9-MeCYP79D1, NC is a no template control. For (B) NC is wild-type control.

**Supplementary material 7:** Chromatogram data

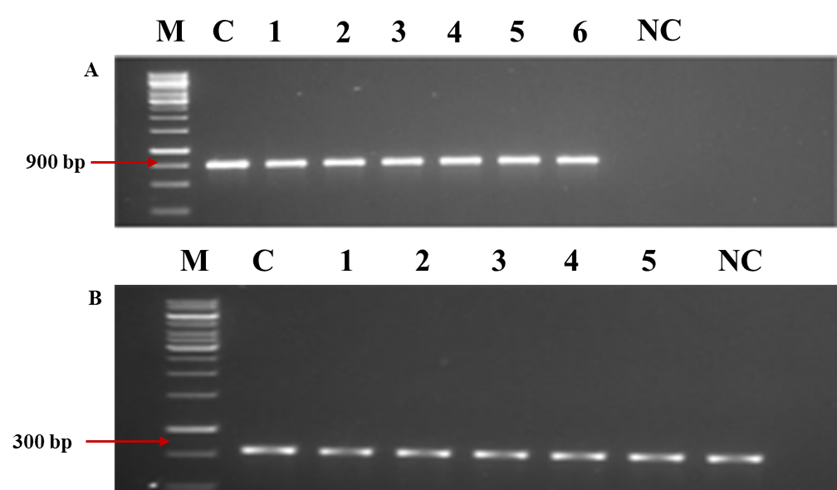


**Supplementary material 8:** RT-PCR analysis of putative TMS 60444 cassava transgenic lines; A: RT-PCR analysis using Cas9 specific primers; B: PT-PCR analysis using ACT specific primers; C, positive control (Plasmid); NC, negative control (non-transformed regenerated plant); M is molecular marker.

**Supplementary material 9:** Linamarin concentration of both transgenic and non-transgenic TMS 60444 cassava

| **Linamarin concentration (g/kg fresh weight)** | | | | | | | | |
| --- | --- | --- | --- | --- | --- | --- | --- | --- |
|  | **Transgenic lines** | | | | | | | |
|  | **TMS 1** | **TMS 2** | **TMS 3** | **TMS 4** | **TMS 5** | **TMS 6** | **TMS 7** | **TMS 8** |
| **Average** | 0.71±0.003 | 0.71±0.002 | 0.73±0.003 | 0.645±0.003 | 0.666±0.068 | 0.583±0.028 | 0.496±0.001 | 0.624±0.002 |
|  | **Non-transgenic lines** | | | | | | | |
|  | **TMS9** | **TMS10** | **TMS11** | **TMS12** | **TMS13** | **TMS14** | **TMS15** | **TMS16** |
| **Average** | 2.405±0.004 | 2.465±0.045 | 3.143±0.002 | 2.841±0.004 | 2.379±0.008 | 3.062±0.006 | 2.737±0.005 | 2.648±0.002 |

**Supplementary material 10:** Total cyanide content in cassava leaves of both mutant and wild-type TMS 60444 cassava cultivars

| **Cyanide content in cassava leaves** | | | | | | | | |
| --- | --- | --- | --- | --- | --- | --- | --- | --- |
|  | **Transgenic lines** | | | | | | | |
|  | **TMS 1** | **TMS 2** | **TMS 3** | **TMS 4** | **TMS 5** | **TMS 6** | **TMS 7** | **TMS 8** |
| **Average** | 9.98±0.005 | 49.9±0.082 | 19.8±0.005 | 20.2±0.008 | 19.8±0.014 | 9.9±0.033 | 20.2±0.008 | 29.7±0.086 |
|  | **Non-transgenic lines** | | | | | | | |
|  | **TMS9** | **TMS10** | **TMS11** | **TMS12** | **TMS13** | **TMS14** | **TMS15** | **TMS16** |
| **Average** | 199.98±0.002 | 201.96±0.034 | 197.43±0.009 | 209.06±0.006 | 194.73±0.093 | 184.89±0.085 | 208.37±0.004 | 211.76±0.021 |

**Supplementary material 11:** Analysis of the agronomic traits of transgenic TMS 60444 cassava plants

| **Cassava line** | **Plant height (cm)** | **Petiole length (cm)** | **Leaf length/leaf width (cm)** | **No of leaves per plant (cm)** |
| --- | --- | --- | --- | --- |
| Transgenic | 40.00 ^a^ ± 2.31 | 5.69 ^b^ ± 0.38 | 3.30 ^c^ ± 0.08 | 6.37 ^d^ ± 0.38 |
| Wild type | 36.00 ^a^ ± 4.92 | 3.77 ^b^ ± 1.90 | 2.96 ^c^ ± 0.31 | 7.00 ^d^ ± 0.58 |
